# A Novel, Validated Stability-Indicating UPLC Method for the Estimation of Lansoprazole and its Impurities in Bulk Drug and Pharmaceutical Dosage Forms

**DOI:** 10.3797/scipharm.1210-09

**Published:** 2012-12-03

**Authors:** Papanaboina Venkata Rao, Morrisetty Nagendra Kumar, Maram Ravi Kumar

**Affiliations:** 1 Dr. Reddy’s Laboratories Ltd. IPDO, Bachupally, Hyderabad-500072, A.P, India.; 2 Department of Chemistry, J. N. T. University, Kukatpally, Hyderabad-500072, A.P, India.

**Keywords:** Lansoprazole, Validation, UPLC, Stability indicating

## Abstract

A novel, reversed-phase ultra-performance liquid chromatographic method was developed and validated for the determination of the assay and related substances of Lansoprazole (LAN) in bulk drug and capsule dosage forms. The related substances include degradation and process-related impurities. The method was developed using the Waters Acquity BEH C18 column and gradient program with mobile phase A as a pH 7.0 phosphate buffer and methanol in the ratio of 90: 10 (v/v), and mobile phase B as methanol and acetonitrile in the ratio of 50:50 (v/v). Lansoprazole and its impurities were monitored at 285 nm. Lansoprazole was subjected to the stress conditions of oxidative, acid, base, hydrolytic, thermal, humidity, and photolytic degradation and found to degrade significantly under acid and oxidative stress conditions. The degradation products were well-resolved from the main peak and its impurities, proving the stability-indicating power of the method. The performance of the method was validated according to the present ICH guidelines for specificity, limit of detection, limit of quantification, linearity, accuracy, precision, ruggedness, and robustness.

## Introduction

**Fig. 1:**
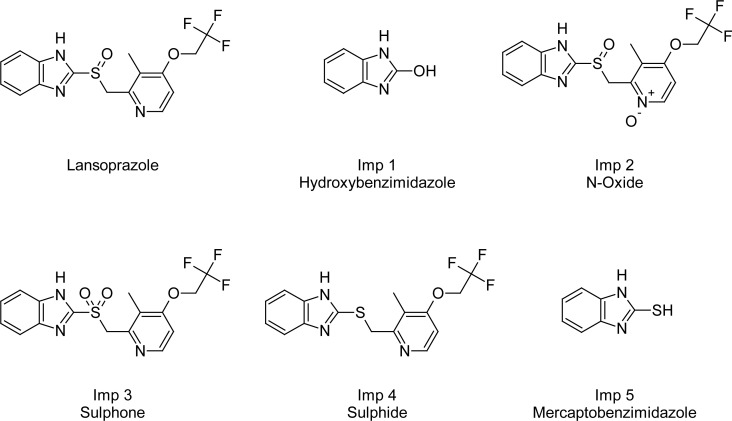
Chemical structures of Lansoprazole and its four impurities

Ultra-performance liquid chromatography (UPLC) is a new category of separation techniques based on well-established principles of liquid chromatography. This utilizes sub-2μm particles for the stationary phase. The UPLC system allows chromatographers to work at higher efficiencies with a much wider range of linear velocities, flow rates, and backpressures. UPLC outperforms traditional HPLC and saves a significant amount of time and cost. In this present work, the technology has been applied for the method development of the assay and related substances procedures of LAN in the bulk drug and capsule dosage form. Lansoprazole is a substituted benzimidazole, named as (*RS*)-2-({[3-methyl-4-(2,2,2-trifluoroethoxy)pyridin-2-yl]methyl}sulfinyl)-1*H*-benzimidazole. Its empirical formula is C_16_H_14_F_3_N_3_O_2_S with a molecular weight of 369.36 (Fig. 1). Lansoprazole is used to treat and prevent stomach and intestinal ulcers, erosive esophagitis (damage to the esophagus from stomach acid), and other conditions involving excessive stomach acid such as Zollinger-Ellison syndrome [1].

In the literature, limited LC methods were reported for the determination of the assay and related substances of LAN. Some analytical assay methods were reported for the estimation of individual and combinations with other drugs in human plasma and dosage forms [2–6]. One chiral method and chemometric approach by HPLC is available to separate known impurities of Lansoprazole, but has not proven the stability-indicating nature [7, 8]. Some research work was done on the synthesis and characterization of impurities formed during the forced degradation of LAN [9, 10]. The present study is on impurities which were formed during the shelf life of bulk drugs and pharmaceutical dosage forms.

A method is available to separate the degradants of Lansoprazole by HPLC with a run time of 60 minutes in the Lansoprazole United States Pharmacopoeia (USP) monograph. The reported USP method was suitable only for bulk drugs and not for pharmaceutical dosage forms. This was evaluated by analyzing the capsules by the USP method, but the LAN and impurity recoveries were not achieved with the USP method. There is no specific, validated stability-indicating method reported for UPLC for both Lansoprazole bulk drugs and pharmaceutical dosage forms. For the determination of LAN and its impurities, a UPLC method was developed which is capable of determining all of the known and possible degradants. The developed method is validated as per ICH guidelines and found to be precise, specific, accurate, robust, and rugged.

## Materials and methods

### Chemicals and reagents

Lansoprazole standard, Lansoprazole API, Imp 1, 2, 3, 4, and 5 standards with a purity of 99.8%, samples were supplied by Dr.Reddy’s laboratories Ltd, Hyderabad, India. Commercially available Prevacid 30mg capsules were used for the dosage form analysis. HPLC grade methanol, ethanol, acetonitrile, and analytical grade potassium dihydrogen phosphate, orthophosphoric acid, triethylamine, borax, edetate disodium, and sodium hydroxide were purchased from Merck, Darmstadt, Germany. HPLC grade water was prepared using the Millipore Milli-Q Plus water purification system, Bedford, MA, USA.

### Chromatographic conditions and equipment

The LC system of Waters Acquity UPLC with photodiode array detector was used for this study and chromatographic separation was achieved on the Acquity BEH-C18 (50 mm × 2.1 mm × 1.7 μm) column. The separation was achieved by gradient elution. Mobile phase A consisted of a mixture of a pH 7.0 buffer (8.0mL of triethylamine (TEA) in 20 mM KH_2_PO_4_ buffer adjusted to pH 7.0 with orthophosphoric acid), and methanol in the ratio of 90:10 (v/v/v), and mobile phase B consisted of a mixture of methanol and acetonitrile in a ratio of 50:50 (v/v). The flow rate of the mobile phase was 0.3 mL/min. The UPLC gradient program (T/%B) was set as 0.01/20, 2.0/30, 5/50,6.0/70,8.5/70,9.5/20, and 11/20. The column temperature was maintained at 40°C, and the injection volume was 3.0 μL. The chromatographic response was monitored at the wavelength of 285 nm.

### Preparation of diluent, standard, and sample solution

The diluent used for the standard and sample preparation consisted of a pH 11.0 buffer and ethanol in the ratio of 80:20 (v/v)(7.6 grams of borax, 1 gram of edetate sodium in 1000 mL water, adjusted the pH to 11.0 with a diluted sodium hydroxide solution). A standard stock solution of LAN 400 μg/mL was prepared by dissolving the appropriate amount of drug in the diluent. Working solutions containing 1.2 μg/mL and 40 μg/mL were prepared from the stock solution and used for the determination of related substances and for the assay determination respectively. Individual impurity stock solutions were prepared, diluted, and mixed to get 1.2 μg/mL, which was used for the method validation of LAN.

An accurately weighed portion of Lansoprazole pellets equivalent to 40 mg of Lansoprazole was transferred into a 100 mL volumetric flask. To that was added about 70 mL of diluent, and it was sonicated for 25 minutes by maintaining the sonicator temperature about 25°C, diluted to volume with diluent and used for the determination of related compounds. An aliquot of 1.0 mL of this solution was diluted to 10mL with the diluent, yielding 40 μg/mL solution that was filtered through a 0.22 μm nylon membrane filter and used for the determination of the assay of LAN.

### Method validation

The proposed method was validated as per ICH guidelines [11, 12].

#### Specificity

Specificity is the ability of the method to measure the analyte response in the presence of its potential impurities and excipents. To demonstrate the stability-indicating nature of the method, Lansoprazole capsules were stressed under acid, base, peroxide, heat, water, humidity, and photolytic conditions, and analyzed. Peak purity was evaluated using a PDA detector in the stressed samples. The purity angle was less than the purity threshold limit obtained in all of the stressed samples, and had demonstrated the analyte peak homogeneity. The LAN assay was estimated against the LAN reference standard, and the mass balance (% assay + % impurities + % degradation products) was calculated in all of the stressed samples.

#### Precision

The precision of the related substances was verified by analyzing six individual preparations of LAN samples spiked with 0.30% of its four known impurities, analyzed as per the test preparation, and the %RSD of the area for each impurity was calculated. Similarly, precision of the assay was verified by analyzing six individual preparations of LAN samples at the assay concentration. The intermediate precision of the method for the assay and related substances was also evaluated on different days using a different column and a different UPLC.

#### Limit of Detection (LOD) and Quantification (LOQ)

The LOD and LOQ for impurities were determined at a signal–to-noise ratio of 3:1 and 10:1 respectively, by injecting a series of dilute solutions with known concentrations. Precision was also carried out at the LOQ level by injecting six individual preparations of impurities and the %RSD of the each impurity area was calculated.

#### Linearity

Linearity test solutions for the related substances method were prepared by diluting the impurity stock solutions to the required concentrations. The solutions were prepared at six concentration levels from the LOQ to 150% of the specification level (LOQ, 0.075, 0.15, 0.30, 0.45, and 0.60%). Linearity test solutions for the assay method were prepared from the Lansoprazole stock solution at six concentration levels from 50 to 150% of the assay analyte concentration (i.e. 40μg/mL). The peak area versus concentration data were treated by least-squares regression analysis.

#### Accuracy

The accuracy study was carried out in triplicate at four concentration levels (LOQ, 50%, 100%, and 150%). Standard addition and recovery experiments were conducted on capsules to determine the accuracy of the related substances method. The accuracy of the assay method was performed in triplicate at three concentration levels (20μg/mL, 40μg/mL, and 60μg/mL) by analyzing the capsules as per the test method.

#### Robustness

To determine the robustness of the developed method, experimental conditions were deliberately changed and the tailing factor for Lansoprazole and its impurities were recorded. The flow rate of the mobile phase was 0.3ml/min to study the effect of flow rate on the resolution; the flow was changed by ± 0.03 units. The effect of pH was studied at pH 6.8 and 7.2 instead of 7.0. The effect of the column temperature on the resolution was studied at 35°C and 45°C instead of 40°C.

#### Solution stability

Sample solutions for the estimation of the assay and related substances were prepared and stored in tightly capped volumetric flasks at room temperature and evaluated at different time intervals up to 24 h along with the standard solutions.

## Results and Discussion

### Method Development and Optimization

The main target of the chromatographic method was to achieve the separation between impurities (process-related and degradants) and the main component, LAN. Lansoprazole structure contains a benzimidazole ring with pka value 8.84 and is highly sensitive in acidic conditions. The maximum absorption wavelength of the reference drug solution, processed by-products, and forcibly degraded drug solutions is 285nm, which is the intersecting value obtained from the UV absorption spectra; hence, 285nm was selected as the detection wavelength for LC analysis. Initial method development started to separate all of the degradants of Lansoprazole, where the mobile phase pH was selected based on the pka value and the diluent pH was selected on the basic side by considering that Lansoprazole degrades in acidic conditions. The mobile phase was selected for development, with a buffer containing 20mM KH_2_PO_4_ and 8 ml of triethylamine in 1000 ml of Milli-Q water, and the pH was adjusted to 7.0 using orthophosphoric acid and methanol as organic solvents. Method development trials were initiated with an isocratic method and were able to separate all the known impurities related to Lansoprazole, but not the degradant peak formed during the acid degradation at RRT 1.05. As the isocratic method was not providing adequate selectivity, the gradient method contained 20mM of KH_2_PO_4_ with pH 7.0 and methanol in the ratio of 90:10 (v/v) as mobile phase A and 100% methanol as mobile phase B respectively. A change in the strength of the salt (buffer) in the aqueous phase exerted imperceptible effects on the retention time, resolution, and peak shape of the drug, while the use of acetonitrile in different ratios (30–80%) resulted in peak tailing proportional to the acetonitrile concentration. Replacing acetonitrile with methanol resulted in peak fronting. Hence, a mixture of acetonitrile and methanol was employed to obtain the optimum peak shape. Some trials were performed to check the retention behavior and to get a Gaussian peak on different column stationary phases. Compared to the Acquity BEH C8 and BEH shield columns, the resolution and peak shape was satisfactory in the C18 stationary phase. The Acquity BEH-C18 (50 mm × 2.1 mm × 1.7 μm) was selected as a suitable column for better separation. The effect of pH on the retention behavior of LAN and its impurities was verified with trials performed at pH 7.0, 8.0, and 9.0. As there is no effect on the retention behavior of LAN with the variation of pH, by considering the column stability and its lifetime, pH 7.0 was selected as the mobile phase buffer. The eluted analyte retention time was approximately 4.0 min. Several preliminary chromatographic runs were performed to investigate the suitability for drug content estimation and cost because of the increasing importance of rapid economic analysis in pharmaceutical analysis to increase the throughput. The system suitability parameters were evaluated for LAN and its four impurities. The USP tailing factor for all four impurities and LAN was found to be less than 1.4. The USP resolution of LAN and the four potential impurities were greater than 2.0 between all pairs of compounds.

### Method validation

#### Results from forced degradation studies

**Fig. 2:**
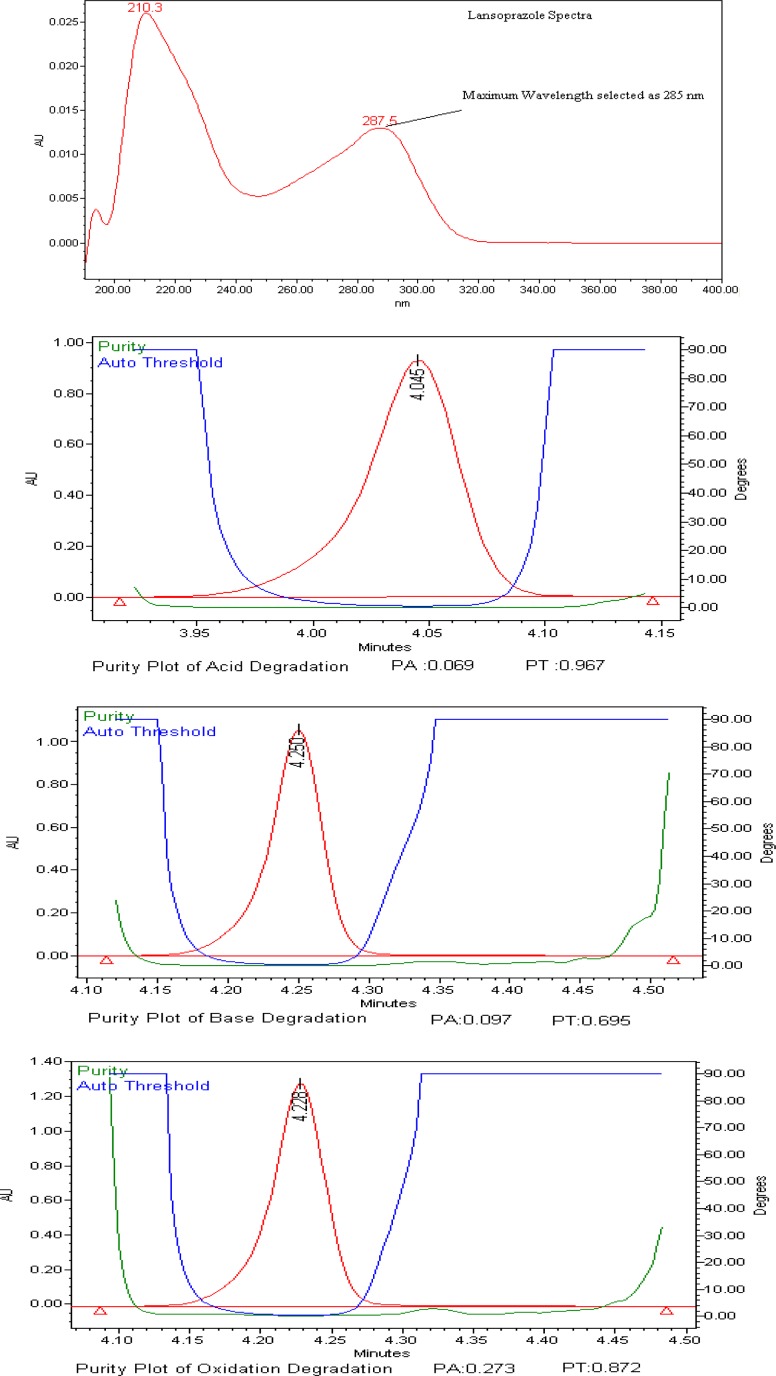
Spectra of Lansoprazole and Peak purity plots

The stress conditions used for the degradation study of Lansoprazole includes heat (105 °C for 14 hours), acid hydrolysis (1N HCl, 1 minute), basic hydrolysis (1N NaOH at 30 °C for 6 hours), aqueous hydrolysis at 60 °C for 30 minutes, oxidation (6% H2O2 for 2 hours), humidity (25°C/90% RH), and visible light (1.2 million lux-hours). Degradation was observed when Lansoprazole was subjected to acid, base, and peroxide degradation conditions; degradation was not observed when subjected to water, humidity, heat, and photolytic conditions. The peak purity of LAN was passed for all of the degradation conditions that showed the homogenous peak of LAN (Fig. 2). Assay studies were carried out for the stress samples against an LAN-qualified reference standard. The mass balance (% assay + % impurities + % degradation products) results were calculated for all of the stressed samples and found to be more than 95% (Table 1). The purity and assay of LAN was not affected by the presence of its impurities and degradation products, which confirms the stability-indicating power of the developed method.

#### Precision

The repeatability of the test method for related substances was checked by a sixfold analysis of 400 μg/mL of Lansoprazole spiked with 1.2μg/mL of each known impurity. The RSD (%) of peak area was calculated for each impurity. The %RSD of Lansoprazole for the method‘s precision was 0.6% and 0.8% for intermediate precision in the assay method. The %RSD of the area for each impurity was calculated for both precision and intermediate precision and was found within 2%. These results confirmed the precision and ruggedness of the method (Table 2).

#### Limits of Detection and Limit of Quantification (LOD & LOQ)

The limit of detection (LOD) is the lowest amount of analyte which can be detected, but not necessarily quantitated as an exact value; the limit of quantification is the lowest amount of analyte which can be determined with suitable precision and accuracy. Both LOD and LOQ values were determined using the signal-to-noise ratio method. The analyte concentration at which the S/N value is around three was considered as the LOD and ten considered as the LOQ. The LOD and LOQ values of Lansoprazole and related substances are given in Table 2.

#### Accuracy

**Fig. 3:**
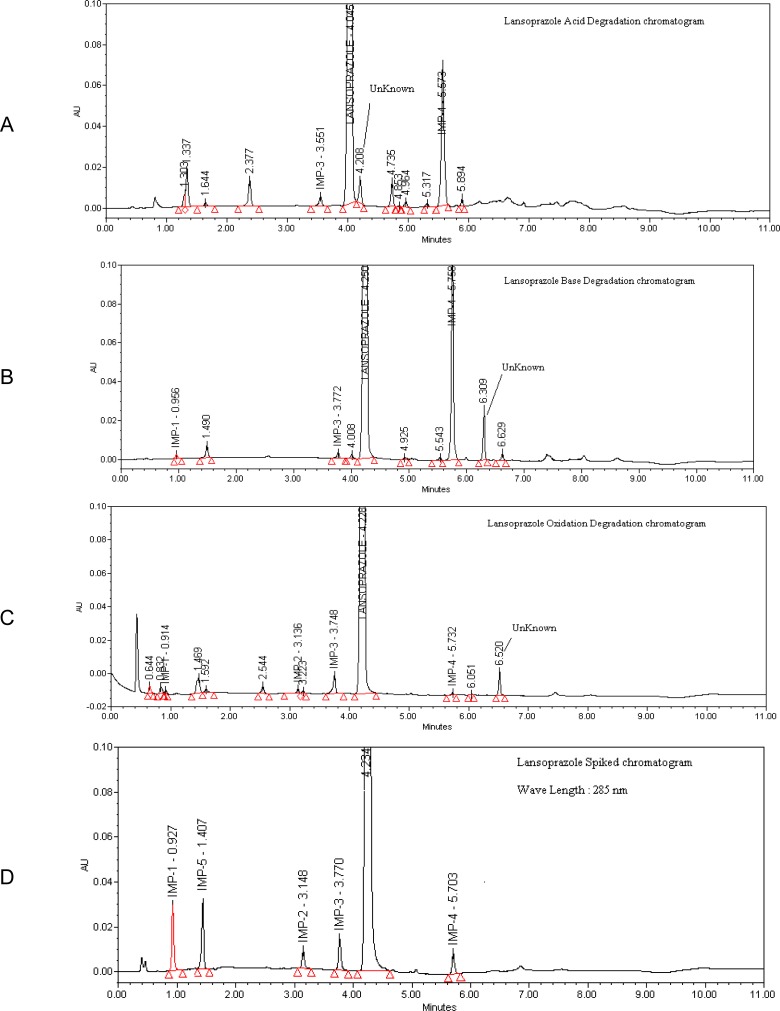
Degradation and Impurity Spiked Chromatograms of Lansoprazole from A to G. A: Lansoprazole Acid degradation chromatogram, B: Lansoprazole Base degradation chromatogram, C: Lansoprazole Oxidation Degradation chromatogram, G: Lansoprazole Spiked chromatogram.

The accuracy of an analytical procedure expresses the closeness of agreement between the reference value and the value found after experimentation. The recovery of impurities was determined in triplicate from the LOQ to 0.6% of the Lansoprazole test concentration. The recovery of Lansoprazole from pharmaceutical dosage forms ranged from 97.8 to 102.6%. The recovery of Lansoprazole impurities from pharmaceutical dosage forms ranged from 93.5 to 106.5%. A UPLC chromatogram for the estimation of related substances obtained from a sample of Lansoprazole spiked with its impurities at 0.3% (1.2 μg/ml) of LAN concentration is shown in Fig. 3.

#### Linearity

The linearity of an analytical procedure is its ability to obtain test results which are directly proportional to the concentration of the analyte in the sample. The linearity of related substances test method was established from the LOQ to 150% of the test concentration for Lansoprazole and its related substances. The correlation coefficient obtained for Lansoprazole and its related substances was >0.998 (Table 2). The calibration plot for the assay method was obtained from 50 to 150% of the test concentration, and the correlation coefficient obtained was greater than 0.999. The results show that an excellent correlation existed between the peak area and the concentration of the analyte. This confirmed the linear relationship between the peak areas and concentrations. The results indicate very good linearity.

#### Robustness

The robustness of an analytical procedure is a measure of its capacity to remain unaffected by small, but deliberate variations in method parameters and provides an indication of its reliability during normal usage. In all of the varied chromatographic conditions (flow rate, column temperature, and pH of the mobile phase), all analyte peaks were adequately separated and there was no change in the elution order of Lansoprazole and its impurities. The effects of variation in chromatographic conditions were also studied in the acid degradation sample in the view of closely eluted degradation peaks, and there was no effect on the resolution of the closely eluted impurities in the sample.

#### Stability in solution and in the mobile phase

Solution stability was established by injecting the same standard, spiked sample, and assay sample at the time intervals of 0 h, 12 h, and 24 h. The standard, spiked sample, and assay sample solution were kept on the benchtop during the study. Mobile phase stability was established by injecting the freshly prepared standard and spiked sample at the time intervals of 0 h, 12 h, and 24 h, without changing the mobile phase lot. No significant changes in the amounts of the seven impurities were observed during the solution stability and mobile phase stability experiments when performed using the related substances method. The difference in assay values of LAN during solution stability and mobile phase stability experiments was less than 1.5%. The results from the solution stability and mobile phase stability experiments confirmed that standard solutions and samples were stable for up to 24h; the mobile phase was stable up to 24h.

## Conclusion

The rapid, gradient RP-UPLC method was developed and validated for the estimation of related substances and the assay in pharmaceutical dosage forms of Lansoprazole. The developed method is specific, precise, accurate, linear, and robust. Satisfactory results were obtained from the validation of the method. The total run time of the method is 11.0 minutes, within which all of the impurities of Lansoprazole are well-separated. The novel UPLC method reduces the analysis time, cost, and exhibits excellent performance in terms of sensitivity and speed. The method can indicate stability and can be used for the routine analysis of production samples and to check the stability of Lansoprazole samples.

## Figures and Tables

**Tab. 1.** Lansoprazole Forced degradation data in all conditions

**Parameter**	**Lansoprazole**	**Imp 1**	**Imp 2**	**Imp 3**	**Imp 4**	**Imp 5**	**% net Degradation**	**Mass Balance**
Base	99.42	Nil	0.36	Nil	0.08	Nil	0.58	99.4
Acid	83	Nil	16.9	Nil	Nil	Nil	17	98.8
Heat	99.9	Nil	Nil	Nil	0.02	Nil	0.1	99.9
Water	99.2	Nil	Nil	Nil	0.07	Nil	0.8	99
Peroxide	99.9	Nil	Nil	Nil	0.08	Nil	0.1	99.9
Sunlight	99.9	Nil	Nil	Nil	0.02	Nil	0.1	99.5
Humidity	99.9	Nil	Nil	Nil	0.01	Nil	0.1	99.5

**Tab. 2.** LOD, LOQ, regression, and Precision data:

**Parameter**	**Lansoprazole**	**Imp 1**	**Imp 2**	**Imp 3**	**Imp 4**	**Imp 5**
LOD %	0.008	0.006	0.007	0.01	0.01	0.009
LOQ %	0.03	0.02	0.02	0.025	0.03	0.02
Regression equation						
Slope (b)	101127.6	52055.5	92384.8	62139.4	62021.1	58918.2
Intercept (a)	–237.3	278.5	319.5	307.3	260.8	552.8
Correlation Coefficient	0.999	0.999	0.999	0.999	0.999	0.999
Precision (%RSD)	0.2	0.6	0.2	1.8	0.4	0.2
Intermediate Precision (%RSD)	0.3	0.2	2.3	0.4	0.8	0.6
